# Chicago Hospital for Women and Children

**Published:** 1879-03

**Authors:** Mary H. Thompson


### Clinical Reports.

ARTICLE VI.

### CHICAGO HOSPITAL FOR WOMEN AND CHILDREN.

(Reported by MARY H. THOMPSON, M.D.)

*Two Cases of Placenta Prævia.*

Case No. 1. Miss E. B., American, single, primipara, was brought to the hospital about noon, Sept. 10, 1878. She was in the first stage of labor, and having considerable hæmorrhage. She said she had flowed all the morning, and that she had felt no movements during the two weeks previous to her entrance since she was injured by a kick upon the abdomen. She thought herself only in the eighth month of pregnancy.

Upon examination by the resident physician, the os uteri was found dilated about one and a half inches in diameter at the time of a pain, and occluded by a spongy mass, with what was thought to be the fœtal head behind it. She was ordered an enema, and when at stool one-half pint of clots was passed from the vagina in addition to the continuous flow. This was followed by a chill and the cessation of the hæmorrhage for an hour, when another half pint of clots was lost.

At four o'clock p. m., I was summoned and found the patient pale and the lips blanched to near the color of the face. The os uteri was about one-fourth dilated, and closed by the placenta, which was so thin upon one side that I was sure it was the circumference and that we had a case of partial placenta prævia. Through the placenta I felt the fœtal head. The flow was continuous, but more profuse between the pains.

The rule by which to be guided in the present case seemed to be to dilate the os uteri as soon as possible, without detriment to the uterus or neighboring tissues, and to remove the fœtus. The question was, how shall it be done?

I believe the greater part of the hæmorrhage in placenta prævia comes from the uterine vessel, and that it is this portion of the hæmorrhage which imperils the woman's life rather than that from the placental vessels. There are always doubts in regard to our power of saving the life of the child, especially if hæmorrhage has existed at different periods during the last months of pregnacy. But if we save the child's life it is by saving the mother's, and terminating labor in the expeditious way consistent with this physiological process of child-birth. Hence, I determined to compress the uterine vessels in the lower zone of the organ, and if possible those of the placenta.

I inserted the tips of three fingers, in the form of a cone, into the os uteri, and spread them, detaching the placenta as far as it could be reached entirely around the margin. The circumference of the placenta being easily reached on one side, was torn and pushed back. The fingers were quickly removed, and pressure from the vaginal surface applied with the hand until after the first contraction. The fingers were again inserted and spread so at to keep the os distended or stretched out, with all the power that could be applied in such a position; but when contractions came on, the fingers were removed and rested during the time, and immediately reinserted.

The blood vessels were thus effectually closed for the time; and soon the labor pains, which were at first weak, became stronger.

To be positive that by this maneuver we were arresting the hæmorrhage, the fingers were removed twice during the periods between "pains," when the hæmorrhage began as at first.

About 5:15 p. m., the first stage terminated, and fluid extract of ergot was given in two gram doses until three doses were given. The pains were stronger for a short time, then grew so weak that the labor was terminated with forceps.

The child had been dead for some time. The cuticle was denuded in some places, and in others it hung in folds, having partially lost its connection with the body.

The mother made a slow but sure recovery.

Case No. 2. Mrs. L. H., aged 40, American, mother of four children, entered the hospital Sept. 18th, 1878. She had slight hæmorrhage and said she was near her "term;" also that she had never bled before when carrying her children. She had flowed four months previous to her entrance and at every succeeding period, also whenever she worked (as she at times was obliged to). The last time was five days ago, after ironing a little, but it ceased after lying down. On the second day after admission, and after walking about the ward, hæmorrhage came on and lasted three hours, ceasing as suddenly as it began. On the 22d, or fourth day after entrance, about four o'clock a. m., slight labor pains began and continued until eleven a. m., with only a little show, but at this time hard pains and hæmorrhage commenced.

I found no dilatation and the cervix nearly an inch thick. Had it been thinned we should have used the fingers as in the other case. Instead, we used the tampon and asked advice of Drs. E. Marguerat and J. Bartlett. They advised the use of the tampon until dilatation was effected. We made the tampon of an ordinary roll of bandage, unwinding a part, then inserting the whole, adding cotton until the vagina was full. But I soon found dilatation of the vagina going on, and hæmorrhage beginning again, unless the whole mass of cotton was pressed against the os uteri. This act not only seemed to control the bleeding, but stimulated contractions.

About half past two o'clock p. m., the tampon was removed and the colpeurynter used instead, but for a short time, however, as it was expelled after being distended to its extreme limit. The tampon was again used after an examination, which confirmed the diagnosis of central placenta prævia, for the os uteri was not only occluded by this thick, spongy body, but a portion protruded into the cervix.

At about four o'clock p. m., Dr. Marguerat returned, when I removed the tampon and by examination found the os sufficiently dilated to admit my hand. Dr. M. advised turning at once. I pushed my hand through the os uteri and placenta and brought down the feet, but then discovered that I had a full-termed fœtus to remove through the small opening in the placenta, and the insufficiently dilated os uteri. I drew down the feet, and continued making traction, hoping the body of the child would enlarge the opening in the placenta, and that in the meantime the cervix would dilate.

The opening in the placenta was made larger, for it was torn into the membranes on one side, and the os was dilated (not lacerated), but the child gave not the least sign of respiration, though the heart beat in a fluttering way for nearly an hour.

In this case, as in the first, the mother made a good but slow recovery after the great loss of blood.

I believe that had we waited until sufficient dilatation had occurred, the child's life as well as that of the mother's might have been saved. In fact, I think too frequently the child is sacrificed, and sometimes the mother, by an undue haste to terminate labor, and that if life is not lost, the mother is injured beyond the power of surgical skill to mend.

